# Cost-Effectiveness of Recombinant Versus Live-Attenuated Herpes Zoster Vaccination in China: A Modeling Study Under Self-Paid and National Immunization Scenarios

**DOI:** 10.3390/vaccines14070587

**Published:** 2026-07-01

**Authors:** Wei Zhang, Song Li, Tianming Yan, Congjun Zhou, Xinyi Lei, Cairong Zhu

**Affiliations:** Department of Epidemiology and Health Statistics, West China School of Public Health and West China Fourth Hospital, Sichuan University, Chengdu 610041, China; 480854732@qq.com (W.Z.); 1725070791@qq.com (S.L.); 2054256009@qq.com (T.Y.); 1744807425@qq.com (C.Z.); leixinyi113@163.com (X.L.)

**Keywords:** herpes zoster, PHN, cost-effectiveness, Markov model

## Abstract

Objective: The aim was to evaluate the cost-effectiveness of self-paid vaccination using Zoster Vaccine Live (ZVL) and Recombinant Zoster Vaccine (RZV) in China, as well as their inclusion in China’s National Immunization Program (NIP), and to determine the most cost-effective initial vaccination age under this inclusion strategy. Methods: Based on the ZONA model, a Markov model was developed by incorporating pain severity levels for herpes zoster (HZ) and postherpetic neuralgia (PHN), allowing for dynamic transitions between health states. The model evaluated the cost-effectiveness of six vaccination strategies from a societal perspective. Economic evaluations were conducted using the incremental cost-effectiveness ratio (ICER), quality-adjusted life-years (QALYs), and incremental cost-utility ratio (ICUR). The robustness of the results was verified through sensitivity analysis. Results: In the self-paid scenario, both RZV and ZVL were economically viable for preventing HZ. However, neither was cost-effective in preventing PHN. When included in the NIP, initiating RZV vaccination at age 50 demonstrated dominance (higher quality-adjusted life years and lower costs), with an ICER of −3826 CNY per HZ case prevented, −38,669 CNY per PHN case prevented, and an ICUR of −11,128 CNY per QALY gained. Subgroup analysis indicated that initiating RZV vaccination at age 50 offered the best cost-effectiveness, while age 70 was the most cost-saving option. Conclusions: Generally, RZV demonstrates greater cost-effectiveness compared to ZVL. Incorporating RZV into the NIP, starting at age 70, is the optimal strategy to reduce the burden of HZ and PHN and improve healthcare resource allocation in China.

## 1. Introduction

Herpes zoster (HZ) is an infectious dermatological condition caused by the reactivation of varicella-zoster virus (VZV) dormant in ganglia [1]. Postherpetic neuralgia (PHN), the most common complication of HZ and associated with age, has an incidence exceeding 20% in patients aged 50 years and older [2,3,4]. Beyond the economic burden, PHN can cause neuropathic pain lasting for years, with a profound negative impact on patients’ emotional well-being and quality of life [5,6,7,8]. The therapeutic effect of pharmacological therapy for PHN is limited, but vaccination can effectively prevent it [1,9,10].

Currently, two herpes zoster vaccines are available in China: Recombinant Zoster Vaccine (RZV) and Zoster Vaccine Live (ZVL). Requiring two intramuscular doses, RZV (3200 CNY for two doses) demonstrates 97.2% efficacy in adults aged 50 years and above, providing protection for over a decade [11,12,13]. In contrast, a single subcutaneous dose of ZVL (1380 CNY per dose) is approximately 58% effective in individuals aged 40 years and above [14]. Despite being recommended, the vaccines have low vaccination rates, primarily due to high costs and insufficient public awareness [15,16]. A Shanghai survey revealed that willingness for vaccination was 19% under self-paid scenarios but increased to 74% with insurance coverage [17].

Given constrained health resources, economic evaluation becomes pivotal to informing the inclusion of vaccines in the National Immunization Program (NIP). Owing to differences in healthcare systems, vaccine pricing, and epidemiological profiles, economic assessments of HZ vaccines demonstrate substantial cross-country variation. For instance, a Canadian study found RZV more cost-effective than ZVL across all ages, with both being most economical at age 75 [18]. Conversely, Korean research indicated that although RZV yielded higher quality-adjusted life-years (QALYs), ZVL’s lower cost made it more economically favorable, peaking in cost-effectiveness at age 60 [19]. However, a Swedish study concluded ZVL vaccination was not cost-effective (ICER €200,000) [20]. The applicability of the only relevant study from mainland China is limited, as it relied on foreign vaccine efficacy parameters and an inappropriate vaccination age [21]. The ZONA model is widely applied within a decision tree-Markov framework to simulate HZ progression. Nevertheless, it assumes PHN resolves automatically after one year and ignores pain severity stratification, thus diverging from clinical reality [22].

This study thus aims to develop a more realistic Markov microsimulation model through individual-level simulation, classifying the states of HZ and PHN by pain levels and allowing dynamic transitions between these states. The research will first evaluate the economic value of self-paid RZV versus ZVL vaccination, then explore differences in cost-effectiveness across various initial vaccination ages under the NIP scenario. Through model structure optimization and parameter adaptation, this study will provide valuable decision-making evidence regarding the inclusion of HZ vaccines in China’s NIP, thereby supporting the implementation of Healthy China 2030 initiatives and facilitating optimal allocation of healthcare resources.

## 2. Method

### 2.1. Vaccination Strategy

This study considered six immunization strategies: (1) no vaccination (NV), where individuals receive neither ZVL nor RZV; (2) self-paid ZVL (SPV-ZVL), where willing individuals receive self-paid ZVL vaccination; (3) self-paid RZV (SPV-RZV), where willing individuals receive self-paid RZV vaccination; (4) voluntary self-paid vaccination (V-SPV), where willing individuals can choose between self-paid RZV or ZVL; (5) NIP-ZVL, where ZVL is included in the NIP; (6) NIP-RZV, where RZV is included in the NIP.

### 2.2. Structure of Markov Microsimulation Model

#### 2.2.1. Model Structure

This study constructed a Markov microsimulation model with 12 health states based on the ZONA model framework while referencing the pain grade setting from Moore L’s study and incorporating the natural history of HZ [23]. Specifically, the health states include healthy, recovered health, HZ with no/mild pain, HZ with moderate pain, HZ with severe pain, PHN with mild pain, PHN with moderate pain, PHN with severe pain, ocular complications, otic complications, other complications and dead. The schematic diagram of Markov state transitions is provided in Figure S1.

**Figure 1:**
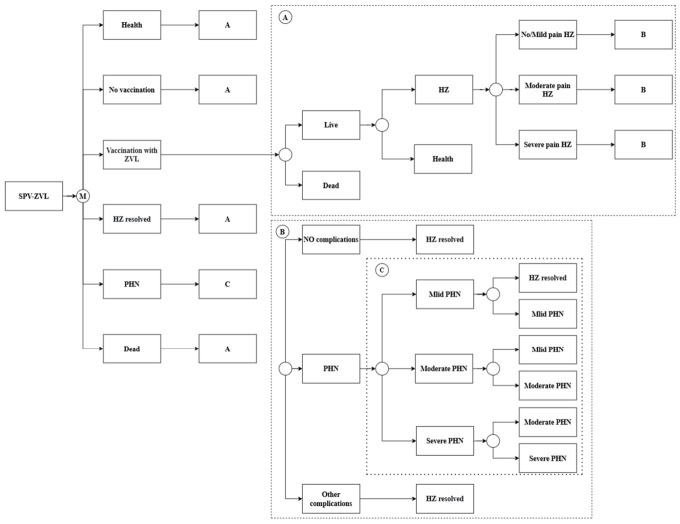
Simplified decision–analytic model (using SPV-ZVL as an example). M = Markov node. Abbreviations: SPV, Self-paid Vaccination; ZVL, Zoster Vaccine Live; RZV, Recombinant Zoster Vaccine; HZ, herpes zoster; PHN, postherpetic neuralgia.

All simulated individuals started in the healthy state. If HZ occurs, patients are transitioned to the corresponding HZ state and categorized by pain grade (no/mild, moderate, or severe). If patients progress to PHN, they are transitioned to the corresponding PHN state. As PHN-related pain eases gradually over time, patients can either maintain the same pain state for multiple cycles or shift to a milder pain state. All PHN patients must go through the PHN with mild pain state to eventually return to the healthy state. Individuals who enter the non-PHN complications state or have no complications will transition to the recovered health state in the next cycle. Recovered individuals can remain in the recovered health state but may experience recurrence with the recurrence pathway matching that of the initial onset, and no restrictions on the number of recurrences were imposed in this study. Study participants may die in any health state, and dead is an absorbing state. Vaccinated individuals who still develop the disease follow the same disease pathway as unvaccinated individuals. Given that HZ typically lasts 2–4 weeks and most PHN pain resolves within a year, a 1-month cycle length was chosen for the model to accurately simulate disease progression, particularly the gradual alleviation of PHN [1,6]. A simplified version of the model is shown in Figure 1.

Clinically, PHN is defined as pain persisting beyond 90 days. In our model, therefore, patient recovery requires the accumulation of at least 3 cycles within the HZ states. For the purpose of tracking PHN duration, all HZ pain states were configured as tunnel states, with tracker variables implemented to tally cycle counts and compute the total PHN time.

#### 2.2.2. Key Parameter Settings

The initial population in the model was randomly sampled from Chinese individuals aged 40–79 years with a total of 10,000 people included. Data on the proportion of individuals in each age group and gender distribution were sourced from the *China Statistical Yearbook 2024*. Given that the average life expectancy of Chinese people reached 78.6 years in 2023, this study set age = 80 as the termination condition for the Markov model—meaning individuals in the simulated population would exit the model when they reached 80 years of age [24]. Data on natural mortality rates were obtained from the *China Cause of Death Surveillance Dataset 2021*. HZ incidence data were obtained from a descriptive analysis of 11,202 cases in Beijing, which reported gender- and age-stratified annual rates subsequently converted to monthly probabilities [25]. Pain severity classification for PHN across all levels was established according to the Shingles Prevention Study [26]. Under the current self-paid vaccination scheme in China, parameter sources for population vaccination coverage, vaccination compliance, and the market distribution ratio between ZVL and RZV are provided in Table S1.

### 2.3. Half-Period Correction

By default, state transition events in Markov models occur synchronously at the end of each cycle, yet state transitions in real populations happen incrementally. This assumption may lead to an overestimation of the expected survival time for absorbing states (such as death) and an underestimation of the expected survival time for non-absorbing states. To correct for this error, the study applied half-cycle correction. Its principle involves assigning half of the benefit value associated with the study subjects’ initial state to the start of the cycle and the other half to the post-transition state at the end of the cycle, thereby enabling more accurate estimation of survival time.

### 2.4. Sensitivity Analysis

Sensitivity analysis is used to assess the impact of parameter uncertainty on results, and this study employed one-way sensitivity analysis and probabilistic sensitivity analysis to verify the stability of the findings. With reference to the results of previous sensitivity analyses on the economic evaluation of HZ vaccines, this study intends to conduct sensitivity analysis on the following parameters: the HZ incidence rate, recurrence rate, proportion of PHN among HZ patients, transition probabilities between different PHN pain grades, HZ vaccine efficacy decay rate, vaccine coverage rate under self-paid vaccination, ratio of ZVL to RZV, price of HZ vaccines under the NIP, direct medical costs of HZ treatment, direct medical costs of PHN treatment, HZ health utility value, PHN health utility value, and discount rate (5%). The value ranges of parameters in the sensitivity analysis and the parameters of the fitted distributions are provided in Tables S2 and S3.

### 2.5. Contents of Economic Assessment

The base-case analysis of this study consists of two parts. First, from a societal perspective, the study will take NV as the comparator strategy to initially explore the economy of the two vaccines in the self-paid vaccination scenario in China. Considering the real-world context of individual choice in the vaccination market, after completing the economic evaluation of self-paid vaccination strategies, the V-SPV will be used as the comparator to assess the cost-effectiveness of NIP-ZVL (250 CNY per dose) and NIP-RZV (600 CNY for two doses). Second, we will perform subgroup analyses stratified by initial vaccination age to explore economic viability across alternative starting ages with the aim of identifying the optimal initial vaccination age.

## 3. Results

### 3.1. Base-Case Analysis

The model simulated 10,000 people 1000 times, and the results are shown in Table 1. Compared with NV, the ICERs for SPV-ZVL were 48,475 CNY per HZ case avoided and 553,314 CNY per PHN case avoided. The corresponding ICERs for SPV-RZV were 16,560 CNY and 167,664 CNY, respectively. At the willingness-to-pay (WTP) threshold, both vaccines were cost-effective for preventing HZ (RZV more so than ZVL), but neither was cost-effective for preventing PHN as their ICERs exceeded the threshold. The QALYs for SPV-ZVL and SPV-RZV were 105,201 and 105,204, respectively. The corresponding ICURs versus NV were 96,406 CNY per QALY for SPV-ZVL and 30,787 CNY per QALY for SPV-RZV. Despite the cost-effectiveness of SPV-RZV’s QALY gain relative to SPV-ZVL, the ICUR values exhibited wide variation across simulations, pointing to significant result uncertainty.

Compared with the V-SPV, the ICERs for NIP-ZVL were 80,573 CNY per HZ case avoided and 301,514 CNY per PHN case avoided. In contrast, NIP-RZV was a dominant strategy, with ICERs of –3826 CNY per HZ case avoided and –38,669 CNY per PHN case avoided. At the WTP threshold, NIP-ZVL was not cost-effective in preventing HZ but may be deemed cost-effective in preventing PHN. This is due to both its incremental cost and incremental effect being negative, as under such conditions only an ICER exceeding the WTP threshold indicates that the strategy is worthwhile. In contrast, NIP-RZV was unambiguously superior, preventing more cases of HZ and PHN at a lower total cost. Relative to the V-SPV, NIP-ZVL and NIP-RZV yielded QALYs of 105,203 and 105,221, with corresponding ICURs of –12,099 CNY and –11,128 CNY per QALY, respectively. Similar to the self-paid scenario, the ICURs in the NIP also showed wide fluctuations.

### 3.2. One-Way Sensitivity Analysis

**Figure 2:**
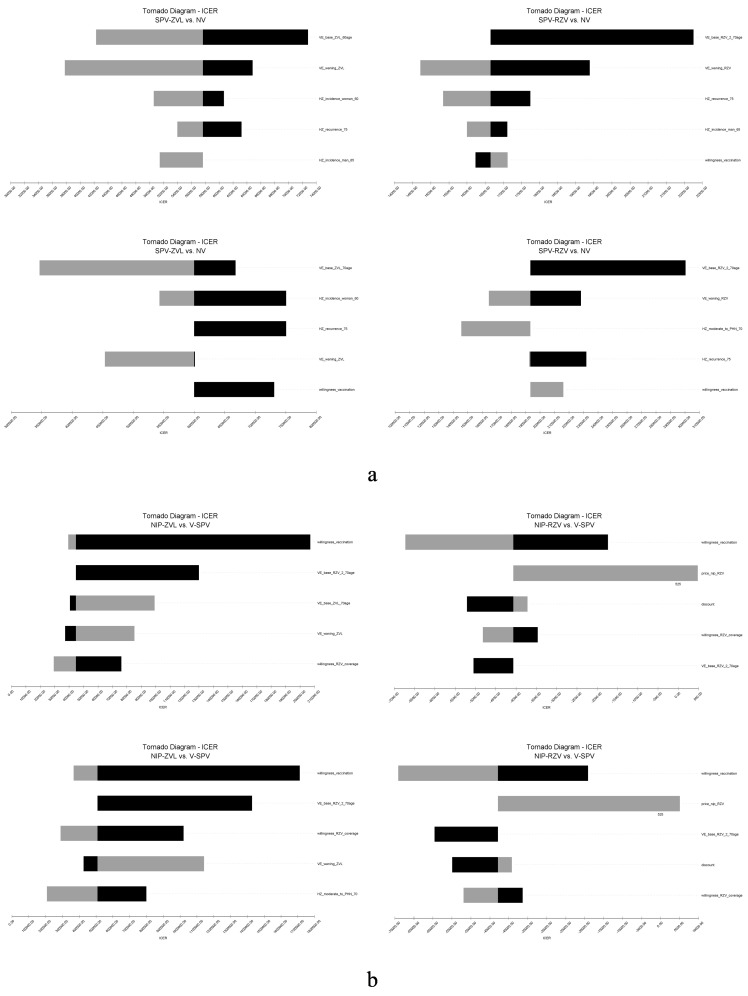
One-way sensitivity analysis of different vaccination strategies. (**a**) ICER tornado diagram for HZ prevention; (**b**) ICER tornado diagram for PHN prevention.

The results of the one-way sensitivity analysis are presented in Figure 2. For SPV-ZVL compared with NV, the most influential parameters on the ICER for avoiding HZ occurrence were ZVL efficacy, ZVL efficacy decay rate and female HZ incidence rate, etc. The key parameters affecting the ICER for avoiding PHN were ZVL efficacy, female HZ incidence rate and HZ recurrence rate, etc. Regarding SPV-RZV, the primary parameters influencing the ICER for avoiding HZ occurrence were RZV efficacy, RZV efficacy decay rate and HZ recurrence rate, etc. The main parameters affecting the ICER for avoiding PHN, however, were RZV efficacy, RZV efficacy decay rate and proportion of PHN among HZ patients, etc.

When vaccines are included in the NIP, for NIP-ZVL compared with V-SPV, the most impactful parameters on the ICER for avoiding HZ occurrence were vaccine coverage under self-paid vaccination, RZV efficacy and ZVL efficacy, etc. The main parameters affecting the ICER for avoiding PHN were ZVL efficacy, vaccine coverage under self-paid vaccination and RZV efficacy, etc. As for NIP-RZV, the parameters that mainly affected the ICER for avoiding HZ occurrence were vaccine coverage under self-paid vaccination, RZV price under the NIP and discount rate, etc. And the key parameters influencing the ICER for avoiding PHN were vaccine coverage under self-paid vaccination, RZV price under the NIP and RZV efficacy, etc.

### 3.3. Probabilistic Sensitivity Analysis

**Figure 3:**
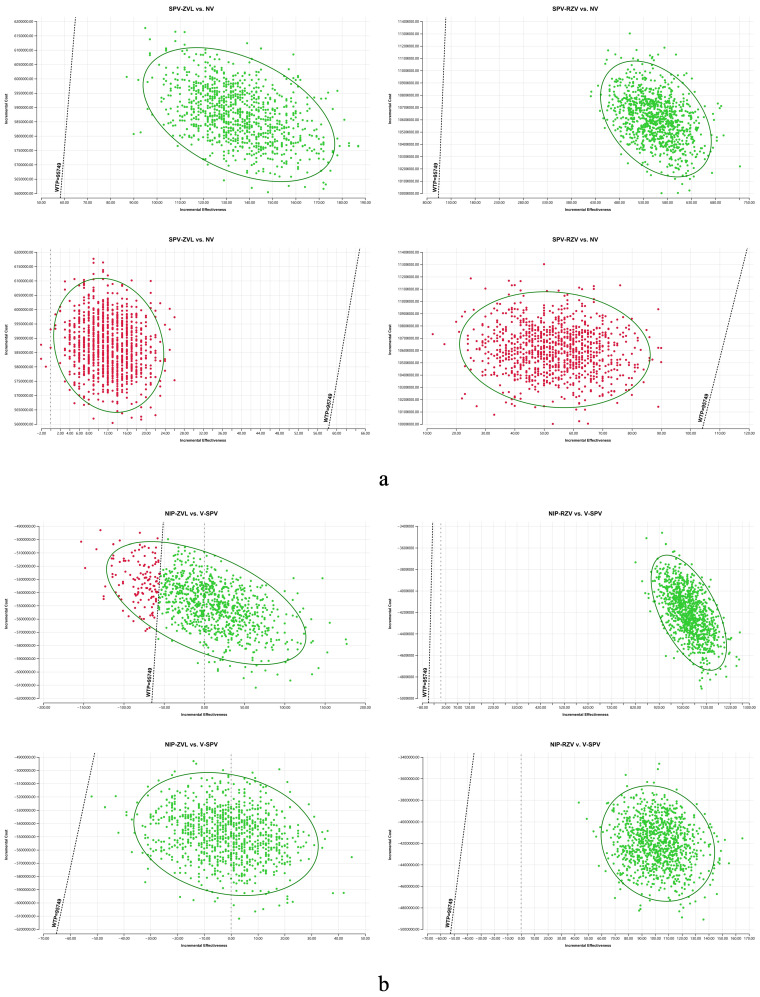
Probabilistic sensitivity analysis of different vaccination strategies. (**a**) ICER scatter plot for HZ prevention; (**b**) ICER scatter plot for PHN prevention.

The results of the probabilistic sensitivity analysis are presented in Figure 3. From the figure, it is evident that when NV was used as the comparator, 100% of the simulations showed that both SPV-ZVL and SPV-RZV were more worthwhile than NV in terms of avoiding HZ occurrence. In terms of avoiding PHN occurrence, 100% of the simulations indicated that neither SPV-ZVL nor SPV-RZV was cost-effective compared with NV. These findings confirm that the results of the base-case analysis are robust.

When compared with V-SPV, regarding the avoidance of HZ occurrence, 53.1% of the simulations showed that NIP-ZVL was a dominant strategy, 33.7% of the simulations indicated that NIP-ZVL was worthwhile, and 13.2% of the simulations demonstrated that NIP-ZVL was not worthwhile. In contrast, 100% of the simulations consistently showed that NIP-RZV was a dominant strategy. When it comes to avoiding PHN occurrence, 47.8% of the simulations revealed that NIP-ZVL was a dominant strategy, and 52.2% of the simulations showed that NIP-ZVL was worthwhile. Again, 100% of the simulations confirmed that NIP-RZV was a dominant strategy. Overall, whether in terms of avoiding HZ or PHN occurrence, including ZVL in the NIP is cost-effective in most cases, while including RZV in the NIP is consistently a dominant strategy. This further validates that the results of the base-case analysis are robust.

### 3.4. Subgroup Analysis of Different Initiation Ages for Vaccination

The economic evaluation results of different initial vaccination ages are presented in Table 2. In terms of avoiding HZ occurrence, the NIP-ZVL with vaccination initiation at age 60 demonstrated superior cost-effectiveness compared to initiation at ages 40, 50, or 70. Relative to the V-SPV, the median ICER was −81,918 CNY per HZ case avoided, indicating the dominance of this approach. For the RZV, incorporation into the NIP with vaccination starting at age 50 was identified as the optimal strategy, yielding an ICER of 8004 CNY per HZ case avoided compared to initiation at age 60 and an ICER of CNY 1996 per case compared to initiation at age 70. With regard to preventing PHN, the most cost-effective strategy for ZVL under the NIP was vaccination initiation at age 60, resulting in an ICER of −306,130 CNY per PHN case avoided relative to the V-SPV, thereby demonstrating complete dominance. For RZV, the optimal strategy involved vaccination starting at age 60, which yielded a median ICER of −39,326 CNY per PHN case avoided compared to initiation at age 50 and an ICER of CNY 8858 per PHN case avoided compared to initiation at age 70.

From a cost-utility perspective, ZVL vaccination initiated at age 50 years resulted in an ICUR of −13,323 CNY per QALY gained compared with the V-SPV, indicating dominance. For RZV, initiation at age 70 years was associated with better cost-utility than initiation at other ages, with ICURs of 3604 CNY and 1779 CNY per QALY gained, respectively.

## 4. Discussion

A systematic comparative analysis of the health and economic effects of ZVL and RZV was performed in the 40–79-year-old Chinese population under self-paid and NIP vaccination scenarios. Although both vaccines reduced the risk of PHN onset in the self-paid vaccination scenario, RZV showed greater advantages in both disease prevention and cost-effectiveness. Compared with NV, the median ICER of RZV for avoiding HZ occurrence is 16,560 CNY per case which is significantly lower than ZVL’s 48,475 CNY per case. At the WTP threshold, RZV is more cost-effective than ZVL for avoiding HZ occurrence. However, neither vaccine was cost-effective for preventing PHN, as their respective ICERs exceeded the WTP threshold. In the NIP scenario with V-SPV as the comparator, RZV demonstrated dominance when vaccination was initiated at age 50. The resulting ICERs for avoiding HZ and PHN were −3826 CNY and −38,669 CNY per case, respectively, demonstrating dominance (lower costs and higher effectiveness) [27]. It was also noted that the price of self-paid vaccination is a primary factor influencing vaccination rates [15]. Within the NIP context, RZV demonstrated significantly higher cost-effectiveness than ZVL among adults aged 40–79, a key factor being its more stable and prolonged protective effect. This was particularly evident in the population aged 50 years and above, a demographic in which RZV demonstrated a balance between health benefits and cost-effectiveness [11,12,13].

The cost-effectiveness of self-paid vaccination with either HZ vaccine in China was found to be limited. Although the ICERs for preventing HZ were below the WTP threshold, those for preventing PHN consistently exceeded it, with the benefit–cost ratio (BCR) being below 1 (Table S4). This conclusion stands in marked contrast to the findings of a United States study, which reported a BCR of 1.57 for vaccination in adults aged 50 years and above [28]. The primary reason for this discrepancy lies in the divergent cost structures. In the United States, the cost of HZ treatment (including productivity losses) is USD 6239.38 (USD/RMB ≈ 6.8) per case, whereas the vaccine price is only USD 140 per dose. In China, however, the treatment cost is approximately 3820 CNY per case, in contrast to the higher vaccine price of 1384–1608 CNY per dose. In addition, self-paid vaccination contributes little to QALYs. One reason is that HZ and PHN are mostly short-term diseases, so the QALY gain from a single vaccination is low. Additionally, clinical variability in the Markov microsimulation model (such as lower expected QALYs for older vaccine recipients and premature death) may obscure the benefits of vaccination. Furthermore, with the model’s endpoint set at 80 years and a cumulative PHN incidence of only 10% by that age, the potential preventive benefits are thus underestimated [29]. However, a study by Wang et al. reported that both HZ vaccines demonstrated cost-effectiveness in improving QALYs, a finding that contrasts with the present results [21]. The discrepancy is primarily attributable to the initiation of ZVL vaccination at the recommended age of 40 years in this study, coupled with the adoption of a more realistic pain grade simulation method.

The economic outcomes can be substantially improved by including HZ vaccines in the NIP, with RZV outperforming ZVL. The ICER of NIP-RZV vaccination is 9870 CNY per QALY at age 50 and decreases to 7650 CNY per QALY at age 70, both of which are below the one-time GDP per capita threshold. Through the National Vaccine Central Procurement System of the Chinese Center for Disease Control and Prevention, large-scale, demand-based bidding can significantly reduce vaccine prices. Even if all eligible elderly people are covered, the total vaccination cost is still lower than the current self-paid situation [30]. At the same time, vaccination can reduce the incidence of HZ, consequently lowering overall societal treatment costs and achieving the dual benefit of saving costs and improving health outcomes. Analysis of the two vaccines indicates that ZVL is cost-effective when administered to the 40–50 age group. Whilst the treatment regimen remains economical despite a slight cost increase for those aged 60–70, RZV is associated with a superior cost-effectiveness profile, demonstrating the advantage of lower cost and greater effectiveness in all age groups. At the policy level, the Vaccine Administration Law of the People’s Republic of China authorizes local governments to expand the range of NIP vaccines based on local needs [31]. Some regions have launched pilot programs to include influenza vaccines in the local NIP, providing a reference for RZV pilot programs [32].

Considering the comprehensive health benefits and financial feasibility, initiating the RZV vaccination at 70 years old is the most appropriate choice after its inclusion in the NIP. While vaccination at age 50 could avert the most HZ and PHN cases, its implementation is limited by financial conditions. With a population of approximately 530 million people aged 50 and above in China, the total procurement amount for NIP vaccines in 2021 was only 3.63 billion CNY. Initiating vaccination at 50 years old would require funds far exceeding the existing budget. In contrast, vaccinating at 70 years old achieves the optimal cost-effectiveness ratio. RZV also maintains stable efficacy in the population aged 70 years and above, whereas the efficacy of ZVL in this age group drops sharply to 18.63%, which further highlights the advantages of RZV vaccination at 70 years old [33]. In addition, the 70-year-old population has a higher risk of developing HZ and PHN, so the health benefits of vaccination are more concentrated in this demographic. The number of people requiring vaccination is also smaller than that when initiating vaccination at 50 years old, which is more in line with China’s current financial affordability. Therefore, considering the limited funds, selecting the age with the highest cost-effectiveness (70 years old) as the initial vaccination age is the most appropriate choice.

The Markov microsimulation model used in this study provides a more practical tool for the economic evaluation of HZ vaccines. This model was constructed at the individual level and utilized Monte Carlo simulation to generate random numbers for simulating health state transitions. The model incorporates probability distributions and conditional functions to govern the dynamic variation in transition probabilities with age and gender. It also utilizes state variables to capture PHN duration and vaccination intervals. This addresses the limitations of traditional cohort models, such as constant transition probabilities, the assumption of population homogeneity, and limited randomness in disease outcomes [34,35]. The model structure was developed with reference to the ZONA framework and further refined by incorporating a pain severity stratification approach derived from UK studies. Specifically, health states for both herpes zoster and postherpetic neuralgia were categorized into no/mild, moderate, and severe pain levels. A stepwise pain resolution pathway was implemented for PHN, and the cycle length was shortened from one year to one month—an adaptation that better aligns with the typical one-month course of herpes zoster and the dynamic pain progression of PHN [22,36,37,38]. In terms of parameter selection, priority is given to Chinese data such as age distribution and incidence rates. Efficacy data for RZV were obtained from Chinese Phase IV clinical trials, while those for ZVL were obtained from the package inserts of domestic vaccines, which enhances the local applicability of the evaluation results [34].

This study has two main strengths. First, it presents methodological innovations. By introducing pain grading and shortening the cycle to 1 month, it optimizes the simulation of disease progression. Most parameters were derived from Chinese data, making the model more relevant to the local context. Second, it evaluates both voluntary self-paid and NIP vaccination scenarios, making it more consistent with China’s actual vaccination situation. The limitations of the study should also not be overlooked. To begin with, some parameters lack Chinese data such as the health utility values of different pain grades for HZ and PHN and the efficacy decay rate of domestic ZVL, but sensitivity analysis has verified the robustness of the results. Relevant studies can be carried out in China in the future to supplement this data. Next, the model assumes constant transition probabilities between different PHN pain grades, an assumption that may not hold in reality. Finally, the model sets 80 years old as the endpoint and does not include the population aged 80 years and above who have a higher risk of HZ and PHN which may underestimate the cost-effectiveness of the vaccines. We also do not compare the economic benefits of the immunization program discussed in the study with those of other forms of public health services that may be associated with economic benefits [39].

## 5. Conclusions

Building on the ZONA model framework, a Markov microsimulation model was developed in this study, incorporating critical refinements including the stratification of pain severity levels for both HZ and PHN, along with the integration of up-to-date vaccine efficacy data derived from the Chinese population. Key parameters such as waning immunity and productivity losses were also integrated into the model. Compared with NV, self-paid immunization with either ZVL or RZV was found to be cost-effective only in preventing HZ cases, although it was not cost-effective in preventing PHN and demonstrated a limited contribution to gains in QALYs. Given constrained fiscal resources, initiating RZV vaccination at age 70 emerges as the optimal policy choice, effectively balancing healthcare resource allocation with targeted health benefits for the high-risk elderly population.

## Figures and Tables

**Table 1:** Cost-effectiveness analysis of HZ vaccination strategies *.

	NV	SPV-ZVL	SPV-RZV	V-SPV	NIP-ZVL	NIP-RZV
HZ Cases (n)	2620	2496	1965	2281	2336	1018
PHN Cases (n)	260	250	195	227	237	103
HZ Incremental Costs	-	600	1080	-	−574	−483
QALYs	105,193	105,201	105,204	105,209	105,203	105,221
Incremental QALYs	-	4.6	4.9	-	1.2	18.9
HZ ICER	-	48,475	16,560	-	80,573	−3826
PHN ICER	-	553,314	167,664	-	301,514	−38,669
ICUR	-	96,406	30,787	-	−12,099	−11,128

* Note: Costs are in 10,000 CNY, ICER in CNY/case and ICUR in CNY/QALY. Abbreviations: NIP, National Immunization Program; ZVL, Zoster Vaccine Live; RZV, Recombinant Zoster Vaccine; HZ, herpes zoster; PHN, postherpetic neuralgia; QALYs, quality-adjusted life years; ICER, incremental cost-effectiveness ratio; ICUR, incremental cost-utility ratio.

**Table 2:** Subgroup analysis results by vaccination age *.

	NIP-ZVL				NIP-RZV		
Initiation Ages	40	50	60	70	50	60	70
HZ Cases (n)	2336	2299	2243	2568	1018	1130	1731
PHN Cases (n)	237	233	221	255	103	100	160
Incremental Costs	−574	−601	−659	−674	−483	−573	−626
QALYs	105,203	105,205	105,223	105,199	105,221	105,223	105,214
Incremental QALYs	1.2	2.1	−13.9	−20.1	18.9	9.4	6.9
HZ ICER	80,573	75,892	−81,918	23,199	−3826	−4992	−11,451
PHN ICER	301,514	293,076	−306,129	219,900	−38,669	−45,247	−93,289
ICUR	−12,099	−13,323	17,659	14,913	−11,128	−11,233	−11,371

* Note: Costs are in 10,000 CNY, ICER in CNY/case and ICUR in CNY/QALY. Abbreviations: NIP, National Immunization Program; ZVL, Zoster Vaccine Live; RZV, Recombinant Zoster Vaccine; HZ, herpes zoster; PHN, postherpetic neuralgia; QALYs, quality-adjusted life years; ICER, incremental cost-effectiveness ratio; ICUR, incremental cost-utility ratio.

## Data Availability

The original contributions presented in this study are included in the article/Supplementary Material. Further inquiries can be directed to the corresponding author.

## Supplementary Materials

The following supporting information can be downloaded at: https://www.mdpi.com/article/10.3390/vaccines14070587/s1, Figure S1: Markov state transition diagram; Table S1: Vaccine-related parameters for herpes zoster vaccines [26,33,40,41]; Table S2: Parameter ranges for one-way sensitivity analysis [25,29,33,40,41,42,43,44,45,46]; Table S3: Parameters of fitted distributions for probabilistic sensitivity; Table S4: Cost-benefit analysis of HZ vaccination strategies [25,29,33,40,41,42,43,44,45,46].
