# Supplementary material for: Cost-Effectiveness of Recombinant Versus Live-Attenuated Herpes Zoster Vaccination in China: A Modeling Study Under Self-Paid and National Immunization Scenarios
Source: Vaccines (Basel). 2026 Jul 1;14(7):587. doi: 10.3390/vaccines14070587 (PMC13417397; doi:10.3390/vaccines14070587)
Supplement: Supplementary file 1 [file vaccines-14-00587-s001.zip › Supplemental Table S1.pdf]

Supplemental Table S1. Vaccine-related parameters for herpes zoster vaccines. *

| Parameter | Value | Source |
|---|---|---|
| Vaccine Efficacy (%) | | 1 |
| RZV (Full 2-dose series) | | |
| Age 50–69 | 100.00 | |
| Age ≥70 | 100.00 | |
| RZV (1 dose only) | | |
| Age 50–69 | 73.04 | |
| Age ≥70 | 100.00 | |
| ZVL | | |
| Age 40–49 | 37.41 | |
| Age 50–59 | 62.72 | |
| Age 60–69 | 64.43 | |
| Age ≥70 | 18.63 | |
| Waning Rate of Efficacy (% per year) | | |
| RZV (2 doses) | 2.545 | |
| RZV (1 dose) | 5.471 | |
| ZVL | 5.471 | |
| Adverse Event (%) | | 2 |
| RZV | 61.9 | |
| ZVL | 23.7 | |
| Parameters under Self-paid Scenario | | 3 |
| HZ vaccine coverage (%) | 44 | |
| Compliance for 2nd RZV dose (%) | 78.69 | |
| Market share between ZVL and RZV (%) | | 4 |
| ZVL | 58.97 | |
| RZV | 41.03 | |
| Parameters under NIP | | Assumption |
| HZ vaccine coverage (%) | 100 | |
| Compliance for 2nd RZV dose (%) | 90 | |

*Notes: HZ: Herpes zoster. RZV: Recombinant zoster vaccine. ZVL: Zoster vaccine live. National Immunization Program: NIP.

## Reference


1. Oxman MN, Levin MJ, Johnson GR, et al. A vaccine to prevent herpes zoster and postherpetic neuralgia in older adults. *N Engl J Med*. 2005;352(22):2271-2284. doi:10.1056/NEJMoa051016

2. Alexandra Echeverria Proano D, Zhu F, Sun X, et al. Efficacy, reactogenicity, and safety of the adjuvanted recombinant zoster vaccine for the prevention of herpes zoster in Chinese adults ≥ 50 years: A randomized, placebo-controlled trial. *Hum Vaccin Immunother*. 2024;20(1):2351584. doi:10.1080/21645515.2024.2351584

3. Cheng C, Yun J, Yi J, et al. A Meta-analysis of vaccination willingness and influencing factors for herpes zoster in adults. J Prev Med Inf. 2025;41(11):1481-1489. doi:10.19971/j.cnki.1006-4028.240408

4. Liu Y, Tan R. Comparison of basic information and market application data of two herpes zoster vaccines. Med Front. 2024;14(20):138-140.
