# Supplementary material for: Cost-Effectiveness of Recombinant Versus Live-Attenuated Herpes Zoster Vaccination in China: A Modeling Study Under Self-Paid and National Immunization Scenarios
Source: Vaccines (Basel). 2026 Jul 1;14(7):587. doi: 10.3390/vaccines14070587 (PMC13417397; doi:10.3390/vaccines14070587)
Supplement: Supplementary file 1 [file vaccines-14-00587-s001.zip › Supplemental Table S2.pdf]

Supplemental Table S2. Parameter ranges for one-way sensitivity analysis. *

Parameter	Range	Source
Male HZ incidence (%)		±25%[1]
Age 40–44	0.031 – 0.051	
Age 45–49	0.027 – 0.045	
Age 50–54	0.032 – 0.053	
Age 55–59	0.043 – 0.071	
Age 60–64	0.061 – 0.101	
Age 65–69	0.074 – 0.123	
Age 70–74	0.083 – 0.138	
Age 75–79	0.083 – 0.138	
Female HZ incidence (%)		
Age 40–44	0.033 – 0.055	
Age 45–49	0.036 – 0.060	
Age 50–54	0.048 – 0.080	
Age 55–59	0.074 – 0.124	
Age 60–64	0.083 – 0.139	
Age 65–69	0.092 – 0.154	
Age 70–74	0.095 – 0.158	
Age 75–79	0.108 – 0.180	
HZ recurrence (%)		95% CI[2]
Age 40–44	0.058 – 0.173	Assumption
Age 45–49	0.058 – 0.173	Assumption
Age 50–54	0.067 – 0.183	
Age 55–59	0.040 – 0.136	
Age 60–64	0.064 – 0.175	
Age 65–69	0.107 – 0.269	
Age 70–74	0.109 – 0.350	
Age 75–79	0.112 – 0.429	
PHN proportion among HZ patients (%)		±25%[3]
Age 40–49	3.78 – 6.30	
Age 50–59	4.71 – 7.85	
Age 60–69	7.73 – 12.89	
Age 70–79	8.52 – 14.20	

Transition probabilities between PHN pain states (%)	15 – 35	Assumption
Two-dose RZV efficacy (%)		95% CI[4]
Age 50–69	85.29 – 100	
Age ≥70	60.90 – 100	
One-dose RZV efficacy (%)		
Age 50–69	55 – 100	Assumption
Age ≥70	50 – 100	Assumption
ZVL efficacy (%)		Package insert*
Age 40–49	0 – 83.89	
Age 50–59	39.56 – 77.70	
Age 60–69	42.54 – 78.67	
Age ≥70	0 – 63.99	
RZV efficacy waning rate (%)	1.77 – 3.32	Fitted
ZVL efficacy waning rate (%)	3.64 – 7.30	Fitted
Vaccine coverage under self-paid scenario (%)	30 – 58	95% CI[5]
Proportion receiving RZV in the private market (%)	30.77 – 51.29	±25%[6]
Price per dose of RZV (Immunization program)	300 – 600	Assumption
Price per dose of ZVL (Immunization program)	250 – 500	Assumption
Direct medical cost per HZ case	2128.71–3547.86	±25%[2,7]
Direct medical cost per PHN case	2031.72 – 3386.20	±25%[2,7]
Health utility value for HZ		±25%[8]
No/Mild pain	0.56 – 0.94	
Moderate pain	0.50 – 0.83	
Severe pain	0.39 – 0.65	
Health utility value for PHN		±25%[9]
Mild pain	0.54 – 0.90	
Moderate pain	0.47 – 0.79	
Severe pain	0.20 – 0.34	
Discount rate (%)	0 – 8	[10]

*Notes: HZ: Herpes zoster. RZV: Recombinant zoster vaccine. ZVL: Zoster vaccine live.

Reference

1. Zhu ZL, Yang JY, Xu Y. Epidemiological characteristics of initia l outpatient cases of herpes zoster in Changping District, Beijing, 2021. B ulletin of Disease Control and Prevention. 2023;38(6):50-53. doi:10.13215/j. cnki.jbyfkztb.2306013

2. Sun X, Wei Z, Lin H, Jit M, Li Z, Fu C. Incidence and diseas e burden of herpes zoster in the population aged ≥50 years in China: Dat a from an integrated health care network. *Journal of Infection*. 2021;82(2): 253-260. doi:10.1016/j.jinf.2020.12.013

3. Jiang W, Li GW, Xu Y, et al. Analysis of epidemiological chara cteristics of herpes zoster in urban areas of Yichang City from 2016 to 20 17 based on the health management big data platform. Chinese Journal of Vaccines and Immunization. 2019;25(4):432-435. doi:10.19914/j.cjvi.2019.0 4.016

4. Alexandra Echeverria Proano D, Zhu F, Sun X, et al. Efficacy, r eactogenicity, and safety of the adjuvanted recombinant zoster vaccine for the prevention of herpes zoster in Chinese adults ≥ 50 years: A randomize d, placebo-controlled trial. *Hum Vaccin Immunother*. 2024;20(1):2351584. d oi:10.1080/21645515.2024.2351584

5. Cheng C, Yun J, Yi J, et al. A Meta-analysis of vaccination will ingness and influencing factors for herpes zoster in adults. J Prev Med Inf . 2025;41(11):1481-1489. doi:10.19971/j.cnki.1006-4028.240408

6. Liu Y, Tan R. Comparison of basic information and market appli cation data of two herpes zoster vaccines. Med Front. 2024;14(20):138-140 .

7. Yang JJ, Pei S, Xu CZ, et al. Direct economic burden of herpes zoster and postherpetic neuralgia in urban population of Yichang City. Ch inese Journal of Viral Diseases. 2020;10(1):75-77. doi:10.16505/j.2095-0136. 2019.0043 8. Gater A, Abetz-Webb L, Carroll S, Mannan A, Serpell M, J ohnson R. Burden of herpes zoster in the UK: findings from the zoster q uality of life (ZQOL) study. *BMC Infect Dis*. 2014;14(1):402. doi:10.1186/ 1471-2334-14-402

9. Van Seventer R, Sadosky A, Lucero M, Dukes E. A cross-sectio nal survey of health state impairment and treatment patterns in patients wi th postherpetic neuralgia. *Age and Ageing*. 2006;35(2):132-137. doi:10.1093 /ageing/afj048

10. Schmader KE, Levin MJ, Chen M, et al. Impact of Reactogenicity After Two Doses of Recombinant Zoster Vaccine Upon Physical Functioning and Quality of Life: An Open Phase III Trial in Older Adults. Newman A, ed. *The Journals of Gerontology: Series A*. 2021;76(3):485-490. doi:10.1093/gerona/glaa127
