# Supplementary material for: Cost-Effectiveness of Recombinant Versus Live-Attenuated Herpes Zoster Vaccination in China: A Modeling Study Under Self-Paid and National Immunization Scenarios
Source: Vaccines (Basel). 2026 Jul 1;14(7):587. doi: 10.3390/vaccines14070587 (PMC13417397; doi:10.3390/vaccines14070587)
Supplement: Supplementary file 1 [file vaccines-14-00587-s001.zip › Supplemental Table S3.pdf]

Supplemental Table S3. Parameters of fitted distributions for probabilistic sensitivity analysis. *

Parameter	Distribution	Source
HZ incidence (%)	Unif.	[1]
HZ recurrence (%)	Unif.	[2]
PHN proportion among HZ patients (%)		[3]
Age 40-49	Beta (α=75, β=1414)	
Age 50-59	Beta (α=123, β=1836)	
Age 60-69	Beta (α=210, β=1827)	
Age 70-79	Beta (α=127, β=991)	
Transition probabilities between PHN pain states	Unif.	Assumption
Herpes zoster vaccine efficacy (%)	Unif.	[4]
Vaccine efficacy waning rate (%)	Unif.	Fitted
Vaccine coverage under self-paid scenario (%)	Unif.	[5]
Proportion receiving RZV in the private market (%)	Unif.	[6]
Price per dose of RZV (Immunization program)	LogN (μ=5.704, σ=0.570)	Assumption
Price per dose of ZVL (Immunization program)	LogN (μ=5.521, σ=0.552)	Assumption
Direct medical cost per HZ case	LogN (μ=7.951, σ=0.795)	[2,7]
Direct medical cost per PHN case	LogN (μ=7.904, σ=0.790)	[2,7]
Health utility value for herpes zoster	Unif.	[8]
Health utility value for PHN	Unif.	[9]
Discount rate	Unif.	[10]

*Notes: Beta distribution：Beta. Lognormal distribution：LogN. Uniform distribution：Unif.

Reference

1. Zhu ZL, Yang JY, Xu Y. Epidemiological characteristics of initial outpatient cases of herpes zoster in Changping District, Beijing, 2021. Bulletin of Disease Control and Prevention. 2023;38(6):50-53. doi:10.13215/j.cnki.jbyfkztb.2306013

2. Sun X, Wei Z, Lin H, Jit M, Li Z, Fu C. Incidence and disease burden of herpes zoster in the population aged ≥50 years in China: Data from an integrated health care network. *Journal of Infection*. 2021;82(2):253-260. doi:10.1016/j.jinf.2020.12.013

3. Jiang W, Li GW, Xu Y, et al. Analysis of epidemiological characteristics of herpes zoster in urban areas of Yichang City from 2016 to 2017 based on the health management big data platform. Chinese Journal of Vaccines and Immunization. 2019;25(4):432-435. doi:10.19914/j.cjvi.2019.04.016

4. Alexandra Echeverria Proano D, Zhu F, Sun X, et al. Efficacy, reactogenicity, and safety of the adjuvanted recombinant zoster vaccine for the prevention of herpes zoster in Chinese adults ≥ 50 years: A randomized, placebo-controlled trial. *Hum Vaccin Immunother*. 2024;20(1):2351584. doi:10.1080/21645515.2024.2351584

5. Cheng C, Yun J, Yi J, et al. A Meta-analysis of vaccination willingness and influencing factors for herpes zoster in adults. J Prev Med Inf. 2025;41(11):1481-1489. doi:10.19971/j.cnki.1006-4028.240408

6. Liu Y, Tan R. Comparison of basic information and market application data of two herpes zoster vaccines. Med Front. 2024;14(20):138-140.

7. Yang JJ, Pei S, Xu CZ, et al. Direct economic burden of herpes zoster and postherpetic neuralgia in urban population of Yichang City. Chinese Journal of Viral Diseases. 2020;10(1):75-77. doi:10.16505/j.2095-0136.2019.0043

8. Gater A, Abetz-Webb L, Carroll S, Mannan A, Serpell M, Johnson R. Burden of herpes zoster in the UK: findings from the zoster quality of life (ZQOL) study. *BMC Infect Dis*. 2014;14(1):402. doi:10.1186/1471-2334-14-402

9. Van Seventer R, Sadosky A, Lucero M, Dukes E. A cross-sectional survey of health state impairment and treatment patterns in patients with postherpetic neuralgia. *Age and Ageing*. 2006;35(2):132-137. doi:10.1093/ageing/afj048

10. Chinese Society of Pharmacoeconomics. Guidelines for Pharmacoeconomic Evaluations in China 2019. Beijing: Peking University Medical Press; 2019.
