# Supplementary material for: Cost-Effectiveness of Recombinant Versus Live-Attenuated Herpes Zoster Vaccination in China: A Modeling Study Under Self-Paid and National Immunization Scenarios
Source: Vaccines (Basel). 2026 Jul 1;14(7):587. doi: 10.3390/vaccines14070587 (PMC13417397; doi:10.3390/vaccines14070587)
Supplement: Supplementary file 1 [file vaccines-14-00587-s001.zip › Supplemental Table S4.pdf]

Supplemental Table S4. Cost-benefit analysis of HZ vaccination strategies*

	NV	SPV-ZVL	SPV-RZV	V-SPV	NIP-ZVL	NIP-RZV
Vaccination cost		632	1197	863	294	597
Treatment cost	421	390	303	355	350	137
Incremental Cost		632	1197		-569	-266
Saved treatment cost		31	117		5	217
BCR		0.050	0.098		-0.009	-0.817

*Notes: All costs are in 10,000 CNY.
