# Supplementary figures and images for: Cost-Effectiveness of Recombinant Versus Live-Attenuated Herpes Zoster Vaccination in China: A Modeling Study Under Self-Paid and National Immunization Scenarios
Source: Vaccines (Basel). 2026 Jul 1;14(7):587. doi: 10.3390/vaccines14070587 (PMC13417397; doi:10.3390/vaccines14070587)

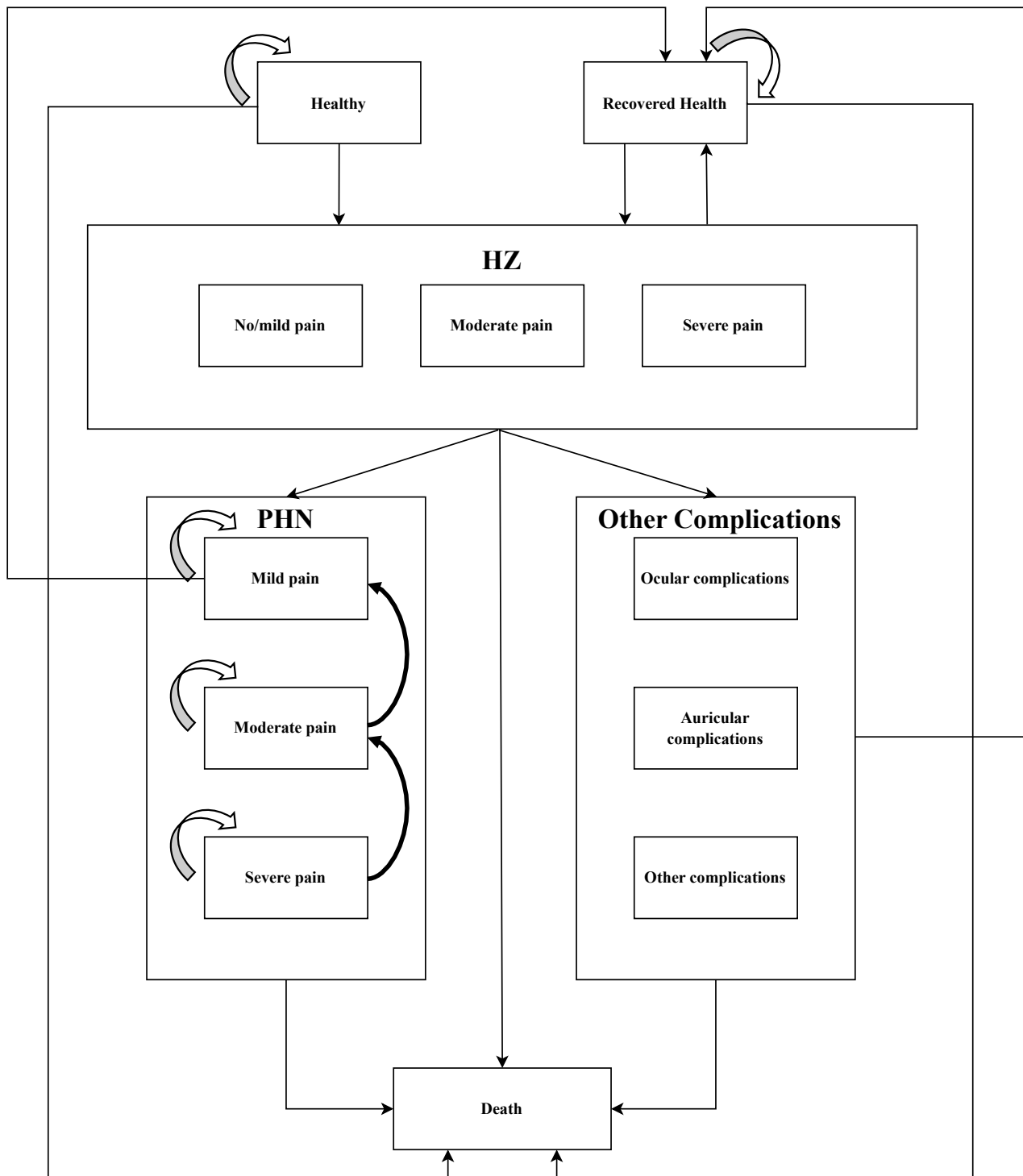

Supplemental Figure S1. Markov state transition diagram.

Supplement: Supplementary file 1 [file vaccines-14-00587-s001.zip › Supplemental Figure S1.pdf]
